# Supplementary material for: Unraveling intertwined orders in the strongly correlated kagome metal CsCr3Sb5
Source: Natl Sci Rev. 2026 Jan 22;13(6):nwag044. doi: 10.1093/nsr/nwag044 (PMC12978309; doi:10.1093/nsr/nwag044)
Supplement: nwag044_Supplemental_File [file nwag044_supplemental_file.pdf]

Supplementary Materials for

Unraveling intertwined orders in the strongly correlated kagome metal $CsCr_3Sb_5$

Liangyang Liu[1,†], Yidian Li[1,†], Hengxin Tan[2,†], Yi Liu[3,4,†], Kuanglv Sun[5,†], Ying Shi[1], Yuxin Zhai[1], Hao Lin[1],

Guanghan Cao[3], Binghai Yan[2], Xianhui Chen[5], Tao Wu[5], Guang-Ming Zhang[1,6*], Luyi Yang[1,7,*]

[1]*State Key Laboratory of Low Dimensional Quantum Physics, Department of Physics, Tsinghua University, Beijing 100084, China.*

[2]*Department of Condensed Matter Physics, Weizmann Institute of Science, Rehovot 7610001, Israel.*

[3]*School of Physics, Zhejiang University, Hangzhou 310058, China.*

[4]*Department of Applied Physics, Key Laboratory of Quantum Precision Measurement of Zhejiang Province, Zhejiang University of Technology, Hangzhou 310023, China.*

[5]*Hefei National Research Center for Physical Sciences at the Microscale, University of Science and Technology of China, Hefei 230026, China.*

[6] *School of Physical Science and Technology, ShanghaiTech University, Shanghai 201210, China.*

[7]*Frontier Science Center for Quantum Information, Beijing 100084, China.*

[*]*E-mails: gmzhang@mail.tsinghua.edu.cn; luyi-yang@mail.tsinghua.edu.cn.*

This file contains the following contents:

1. Experimental setup and pump-induced polarization rotation and reflectivity.

2. Fitting details of the probe polarization and temperature dependence of the time-resolved reflectivity (TRR) data in $CsCr_3Sb_5$.

3. TRR and time-resolved birefringence (TRB) data in $CsCr_3Sb_5$ under the magnetic field.

4. Temperature-dependent TRR data of $CsCr_3Sb_5$ along the principal axis a_2.

5. Fit to the Rothwarf-Taylor model.

6. Coherent phonon oscillations in $CsCr_3Sb_5$ along the principal axis a_1.

7. Anharmonic decay of the coherent phonon mode at 0.88 THz.

8. Calculated low-frequency phonons and their symmetries at the Γ point in the 1×4 swapped antiferromagnetic inverse star-of-David (SA-ISD) CDW phase.

9. Calculated phonon spectra for the normal and CDW phases.

10. Calculation evidence of orbital degeneracy lifting in the ultrafast dynamics.

11. Probe beam wavelength dependence of the TRR and TRB data in $CsCr_3Sb_5$.

12. Fitting details of the probe polarization directions and temperature dependence of the TRB data in $CsCr_3Sb_5$.

13. Fitting details of the critical exponent of the temperature-dependent order parameter.

14. Raman spectra of the $CsCr_3Sb_5$ single crystal.

15. Elastoresistance measurement of $CsCr_3Sb_5$ in the modified Montgomery geometry.

Figures S1–S13.

Table S1

1. Experimental setup and pump-induced polarization rotation and reflectivity.

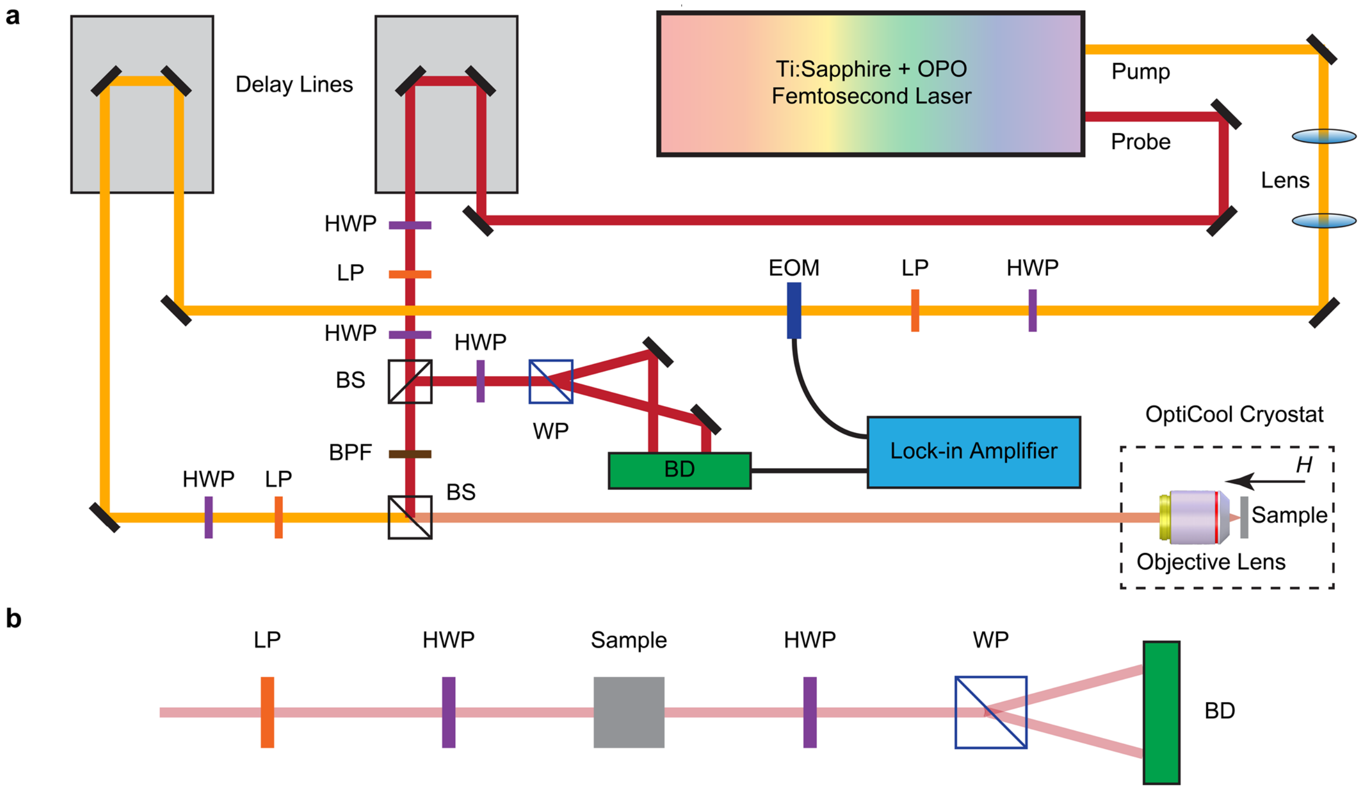

Fig. S1. Lab-based multi-probe ultrafast optical system. (a) Schematic illustration of the experimental setup. (b) Simplified schematic of the setup, containing the elements included in the mathematical description. BS: Beam splitter; LP: Linear polarizer; HWP: Half-wave plate; WP: Wollaston prism; BPF: Band-pass filter; EOM: Electro-optic modulator; OPO: Optical parametric oscillator; BD: Balance detector.

Figure S1a shows the custom-built lab-based multi-probe ultrafast optical system in this study. Detailed descriptions are in the “Methods” section of the main text.

1.1 Static polarimetry

First, we discuss the static polarimetry measurement without pump excitation. This technique measures the change in the angle of linear polarization ($d\phi$) as a function of the input probe polarization (ϕ). A simplified schematic of the setup is shown in Fig. S1b.

We describe the experiment using the Jones matrix formalism. In this framework, the light’s polarization state is represented by a vector in the $\binom{\mathrm{LH}}{\mathrm{LV}}$ basis, and each optical element’s effect is encoded by a 2×2

matrix. For simplicity, we omit the effects of a non-ideal setup that would induce additional polarization rotation (*1*).

The initial polarization is set to be $\begin{pmatrix}1\\0\end{pmatrix}$ by the linear polarizer (LP). The first half-wave plate (HWP), whose fast axis is at θ with respect to the horizontal axis, rotates the polarization of the probe beam by $\phi = 2\theta$. Its Jone matrix is given by $J_{\mathrm{HWP}}(\theta) = \begin{pmatrix}\cos(2\theta) & \sin(2\theta)\\ \sin(2\theta) & -\cos(2\theta)\end{pmatrix}$. The Jones matrix of the sample is given by $J_{\mathrm{Sam}}(r,b,k,\phi_0) = R(\phi_0)J_{\mathrm{Sam}}^{\mathrm{Pr}}(r,b,k)R(-\phi_0)$, where $J_{\mathrm{Sam}}^{\mathrm{Pr}}(r,b,k) = \begin{pmatrix} r+b & k\\ -k & r-b\end{pmatrix}$ is the sample's matrix in its principal axis basis. Here, r represents the isotropic reflection coefficient, b generates birefringence, and k generates the polar Kerr effect, where $b,k \ll r$. $R(\phi_0) = \begin{pmatrix}\cos(\phi_0) & -\sin(\phi_0)\\ \sin(\phi_0) & \cos(\phi_0)\end{pmatrix}$ is the rotation matrix, where ϕ_0 and $\phi_0 + \frac{\pi}{2}$ are the principal optical axes of the sample relative to the lab frame. The fast axis of the second HWP is set at $\theta + \frac{\pi}{8}$ to balance the subsequent detection stage. The resulting polarization state just before the Wollaston prism (WP) is then:

$$\begin{pmatrix}E_H\\E_V\end{pmatrix} = J_{\mathrm{HWP}}\left(\frac{\phi}{2}+\frac{\pi}{8}\right) \times J_{\mathrm{Sam}}(r,b,k,\phi_0) \times\ J_{\mathrm{HWP}}\left(\frac{\phi}{2}\right) \times \begin{pmatrix}1\\0\end{pmatrix}.$$

The WP separates the horizontal and vertical components, and the measured intensity by the balance detector (BD) is given by $I(\phi) = |E_H|^2 - |E_V|^2$. Keeping the terms linear in b,k, the polarization rotation from the sample is given by:

$$d\phi = \frac{I(\phi)}{R} = -\frac{2b}{r}\sin\big(2(\phi-\phi_0)\big) - \frac{2k}{r}, \tag{1}$$

where $R = r^2$ is the equilibrium reflectivity. Rotating the HWPs generates a sinusoidal signal that peaks and bottoms out at $\pm 45°$ relative to the optical axes. This modulation allows us to extract the birefringence (as the amplitude) and the polar Kerr effect (as the polarization-independent offset), as demonstrated in the static optical characterization of CsV_3Sb_5 (*2, 3*).

1.2 Pump-induced polarization rotation

However, the inherent, unvoidable birefringence background in static optical polarimetry poses a challenge. This can be circumvented by employing a pump beam to thermally modulate the order parameters, utilizing either a static or time-resolved measurement approach. In our time-resolved experiments, we measured pump-induced changes in birefringence ($\Delta\theta_B$) and Kerr rotation ($\Delta\theta_K$) as a function of time delay (t) and temperature (T):

$$d\phi(t,T) = \Delta\theta_B(t,T)\sin\big(2(\phi-\phi_0)\big) + \Delta\theta_K(t,T), \tag{2}$$

with $\Delta\theta_B \equiv -2\left(\frac{\Delta b}{r}+\frac{b\Delta r}{r^2}\right)$ and $\Delta\theta_K \equiv -2\left(\frac{\Delta k}{r}+\frac{k\Delta r}{r^2}\right)$, where Δr, Δb and Δk represents pump-induced changes in the isotropic reflection coefficient, birefringence parameter and polar Kerr effect parameter, respectively.

In $CsCr_3Sb_5$, the Kerr rotation effect makes a negligible contribution to the total polarization rotation signal. Specially, when fitting the data (main text, Fig. 4b) to Eq. (2), we found that $\Delta\theta_K$ is only a few percent of $\Delta\theta_B$. This indicates that we do not observe evidence of time-reversal symmetry breaking in $CsCr_3Sb_5$ under our experimental conditions.

1.3 Pump-induced reflectivity

For TRR measurements, we set the second HWP to θ and measure the signal channel only $I(\phi) = |E_V|^2$ (while blocking the balance channel). Following the same Jones matrix formalism procedure, the pump-induced transient reflectivity (normalized by R) is given by:

$$\frac{\Delta R}{R}(t,T) = 2\frac{\Delta r}{r} + 2\left(\frac{\Delta b}{r}+\frac{b\Delta r}{r^2}\right)\cos\big(2(\phi-\phi_0)\big) = 2\frac{\Delta r}{r} - \Delta\theta_B\cos\big(2(\phi-\phi_0)\big). \tag{3}$$

In $CsCr_3Sb_5$, the transient reflectivity exhibits strong anisotropy when rotating the probe polarization at low temperatures (main text Fig. 3d), suggesting $|\Delta r| \ll |\Delta b|$, *i.e.*, the transient reflectivity signal is overwhelmingly determined by the pump-induced change in birefringence (the rotational symmetry breaking order parameter). This observation resembles results found in certain iron-based superconductors (main text Fig. 3f), but sharply contrasts with the nearly isotropic transient reflectivity observed in CsV_3Sb_5 (main text Fig. 3e) (*3*), which implies $|\Delta r| \gg |\Delta b|$.

2. Fitting details of the probe polarization and temperature dependence of the time-resolved reflectivity (TRR) data in $CsCr_3Sb_5$.

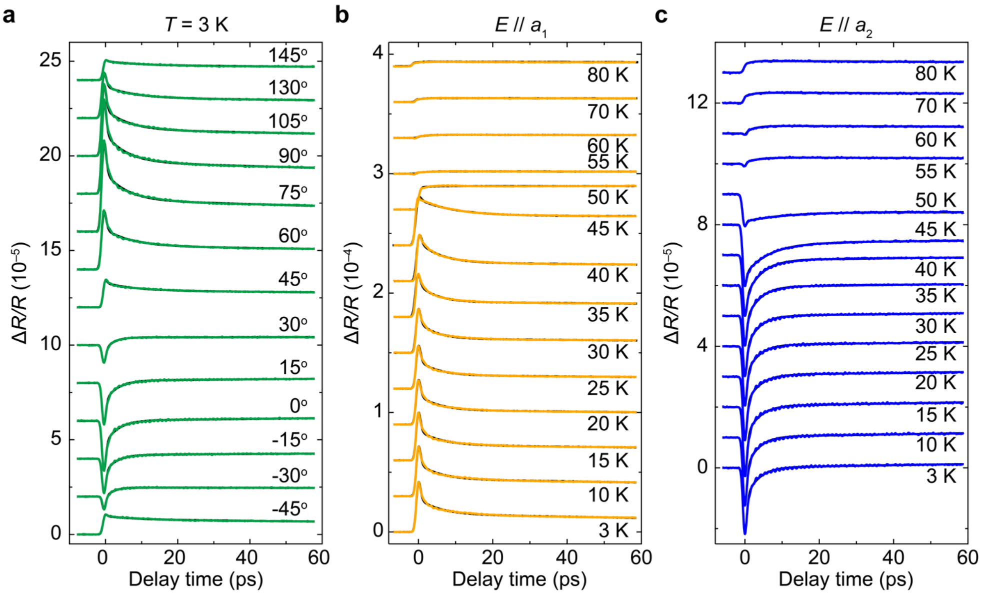

Fig. S2. Fitting details of the time-resolved reflectivity data in $CsCr_3Sb_5$. (a) Probe polarization E dependence of TRR data at $T = 3$ K. (b–c) Temperature-dependent TRR data for probe polarization along the a_1 direction (b) and the a_2 direction (c). All fitting results are overlaid as black lines.

All TRR data are phenomenologically fitted to a double-exponential decay process:

$$\frac{\Delta R(t)}{R} = A_{\text{fast}} \cdot \exp\left(-\frac{t}{\tau_{\text{fast}}}\right) + A_{\text{slow}} \cdot \exp\left(-\frac{t}{\tau_{\text{slow}}}\right) + A_0(t) \qquad (4)$$

where $A_0(t)$ accounts for the residual reflectivity change at long delays, The finite temporal resolution τ_{res} is considered by convolution to a Gaussian function: $\left(1/(\tau_{\text{res}}\sqrt{\pi})\right) \cdot \exp[-(t^2/\tau_{\text{res}}^2)]$.

The amplitudes $A_{\text{fast}}/A_{\text{slow}}$ and the relaxation timescales $\tau_{\text{fast}}/\tau_{\text{slow}}$ can be extracted from the transient reflectivity change with this model. As shown in Fig. S2, this model can nicely fit the probe polarization and temperature dependence of TRR data in Figs. 1e, 2a, 3a–b of the main text. The extracted A and τ are summarized in Figs. 2b–c and Figs. S4c–d.

3. TRR and time-resolved birefringence (TRB) data in $CsCr_3Sb_5$ under the magnetic field.

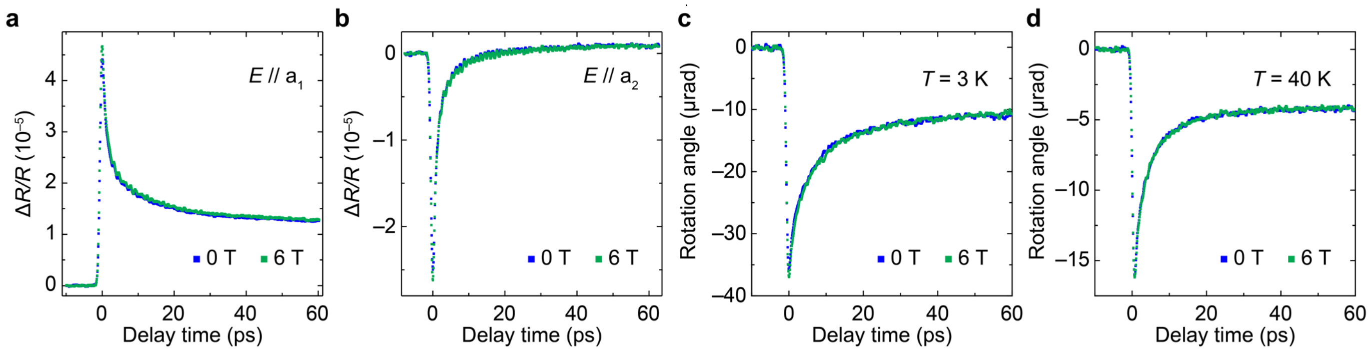

Fig. S3. TRR and TRB data in $CsCr_3Sb_5$ at zero field and under the magnetic field *H* = 6 T. (a–b) TRR data for probe polarization E along the axis a_1 (a) and the axis a_2 (b), data are collected at $T = 3$ K. (c–d), TRB data at $T = 3$ K (c) and $T = 40$ K (d).

Figure S3 compares the TRR and TRB data at zero field and under an applied magnetic field of $H = 6$ T. All the data sets compared at different temperatures and polarizations are almost identical, further substantiating the robustness of the AFM interactions, which remain largely unaffected by the influence of applied magnetic fields. It is noteworthy that the experimental geometry of TRB under the magnetic field is equivalent to the time-resolved magneto-optical Kerr effect (TRMOKE) measurement.

4. Temperature-dependent TRR data of $CsCr_3Sb_5$ along the principal axis $\boldsymbol{a_2}$.

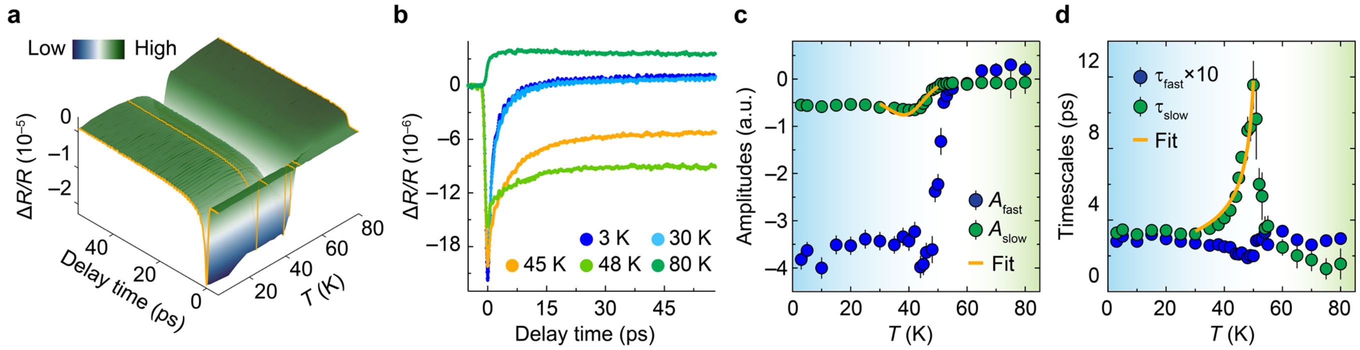

Fig. S4. Temperature-dependent TRR data of $CsCr_3Sb_5$ for the probe beam polarization along the principal axis $\boldsymbol{a_2}$. (a) Three-dimensional plot of the temperature-dependent TRR signals across the phase transition. (b) TRR data at selected temperatures (orange lines in panel a) along the principal axis a_2. (c–d) Temperature-dependent amplitudes (c) and timescales (d) of TRR signals are extracted from the double-exponential fitting of data.

Figure S4 shows the TRR data for the probe beam polarization along the other principal axis a_2. Similar to the data along the axis a_1 in the Figs. 2a–c of the main text, the TRR data along the axis a_2 show a clear phase transition. The extracted temperature-dependent amplitudes A and timescales τ can be described by the Rothwarf-Taylor model, which gives the charge-density-wave (CDW) gap $\Delta_0 = 3.3 \pm 0.7$ meV.

5. Fit to the Rothwarf-Taylor model.

The Rothwarf-Taylor model is usually applied in the ultrafast dynamics in a gapped electronic system (*4-6*). In our work, the temperature-dependent amplitude $A(T)$ and relaxation timescale $\tau(T)$ are described by the following equations:

$$A(T) \propto \frac{F/[\varDelta(T) + k_{\mathrm{B}}T/2]}{1 + \gamma\sqrt{2k_{\mathrm{B}}T/\varDelta(T)}\exp[-\varDelta(T)/k_{\mathrm{B}}T]} \tag{5}$$

$$\frac{1}{\tau} = \frac{12\varGamma_{\omega}k_{\mathrm{B}}T'\varDelta(T)}{\hbar\omega^2} \tag{6}$$

where F represents the fluence of the pump pulse, $\varDelta(T) = \varDelta_0\sqrt{1 - T/T_{\mathrm{c}}}$ is the temperature-dependent energy gap, k_{B} is the Boltzmann constant, $\varGamma_{\omega}$ is the phonon linewidth with frequency ω, T' is the quasiparticle temperature, and $\hbar$ is the reduced Planck constant.

By fitting the extracted temperature evolution of A and τ to the above formulas, we determine the zero-temperature gap value $\varDelta_0$ along two principal axes a_1 and a_2 to be 6.7 ± 2.0 meV and 3.3 ± 0.7 meV, respectively.

6. Coherent phonon oscillations in $CsCr_3Sb_5$ along the principal axis a_1.

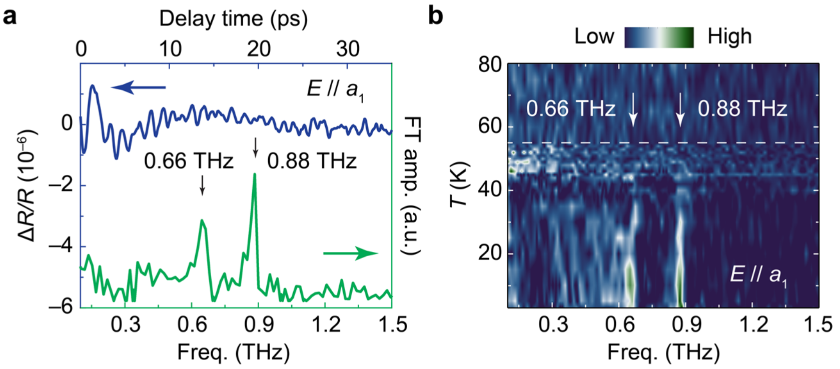

Fig. S5. Coherent phonon oscillations in $CsCr_3Sb_5$ along the principal axis a_1. (a) Residual oscillatory part of the signal (blue) after subtracting the double-exponential background and corresponding Fourier transform (FT) intensity (green) at 3 K along the principal a_1. (b) Temperature-dependent FT intensities map, the white dashed line marks T^*.

Figure S5 presents the coherent phonon oscillations observed for the probe beam polarization aligned along the principal axis a_1. Two distinct phonon modes are identified at frequencies of 0.66 THz and 0.88 THz, which gradually diminish as the temperature rises above T^*. This behavior is consistent with the observations for the a_2 axis shown in Figs. 2d–f of the main text, supporting the temperature-dependent modulation of phonon modes in $CsCr_3Sb_5$.

7. Anharmonic decay of the coherent phonon mode at 0.88 THz.

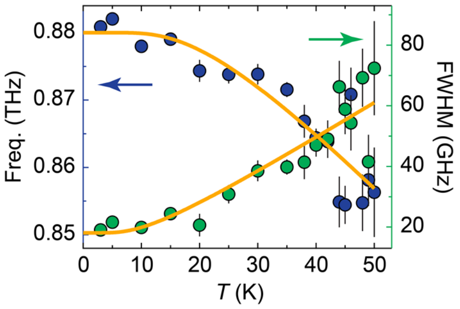

Fig. S6. Temperature-dependent frequency and full width at half maximum (FWHM) of the coherent phonon at 0.88 THz. The solid lines represent fits to the data using the Klemens model.

In Figure S6, the observed softening of the phonon frequency and the broadening of its FWHM linewidth with increasing temperature suggest an anharmonic phonon interaction, consistent with the Klemens model (*7*). This model describes the temperature dependence of phonon frequency $\omega(T)$ and linewidth $\Gamma(T)$ by the following equations:

$$\omega(T) = \omega_0 - A\left(\frac{2}{e^{\hbar\omega_0/2k_B T} - 1}\right) \tag{7}$$

$$\Gamma(T) = \Gamma_0\left(1 + \frac{2}{e^{\hbar\omega_0/2k_B T} - 1}\right) \tag{8}$$

where ω_0 and Γ_0 represent the zero-temperature frequency and linewidth. A is the fitting parameter. k_B and $\hbar$ are the Boltzmann constant and the reduced Planck constant, respectively.

8. Calculated low-frequency phonons and their symmetries at the Γ point in the 1×4 swapped antiferromagnetic inverse star-of-David (SA-ISD) CDW phase.

Index	Frequency (THz)	Symmetry	Raman Active
1	0	B_{3u}	No
2	0	B_{2u}	No
3	0	B_{1u}	No
4	0.47	A_u	No
**5**	**0.59**	$\mathbf{B_{2g}}$	**Yes**
**6**	**0.79**	$\mathbf{A_g}$	**Yes**
7	0.87	B_{2u}	No
8	1.00	A_u	No
9	1.04	B_{3u}	No
10	1.08	B_{3g}	Yes

Table S1. Calculated low-frequency phonons and their symmetries at the Γ point in the 1×4 SA-ISD CDW phase. The phonon modes observed in our experiments are marked in bold font.

Table S1 displays the phonon frequencies, symmetries, and Raman activity of the ten lowest-frequency phonons at the Γ point in the 1×4 SA-ISD CDW phase. Two Raman active phonon Modes 5 and 6, are the most promising candidates in TRR experiments. The calculation is based on an orthorhombic structural approximation, which is a common and justified approach for studying this material (*8-11*).

9. Calculated phonon spectra for the normal and CDW phases.

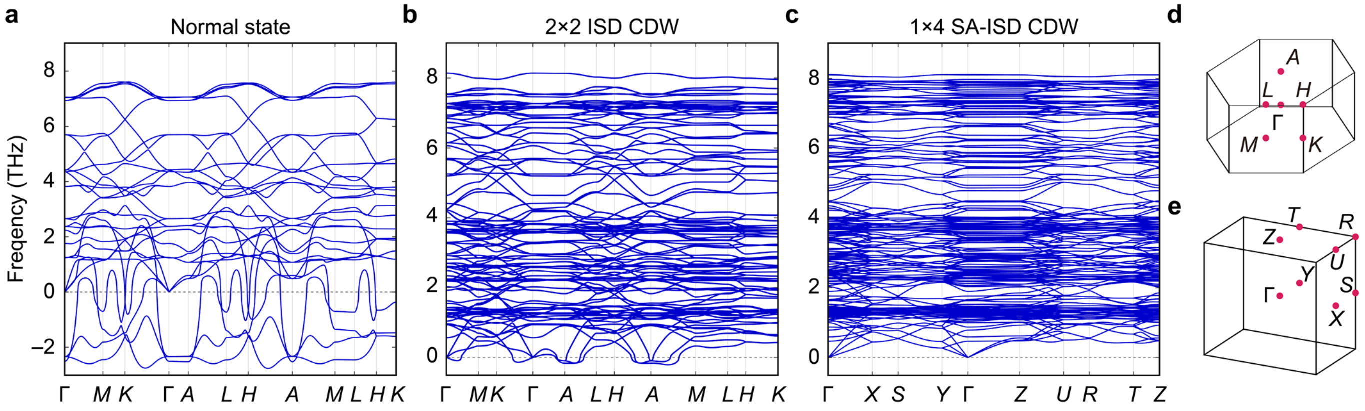

Fig. S7. Calculated phonon spectra for three phases. (a) The normal phase. (b) The 2×2 ISD CDW phase. (c) The 1×4 SA-ISD CDW phase. (d) Brillioun zones (BZ) of the panels a–b. (e) BZ of the panel c.

Figure S7 presents the calculated phonon spectra for the normal phase and two different AFM CDW phases. The imaginary frequency modes in the normal phase and the 2×2 ISD CDW phase suggest their structural instability. The low-frequency CDW amplitude modes we observed only align with the 1×4 CDW structure, which comes from the zone folding of the acoustic phonon branches into the optical phonon branches at the Γ point in the SA-ISD phase.

10. Calculation evidence of orbital degeneracy lifting in the ultrafast dynamics.

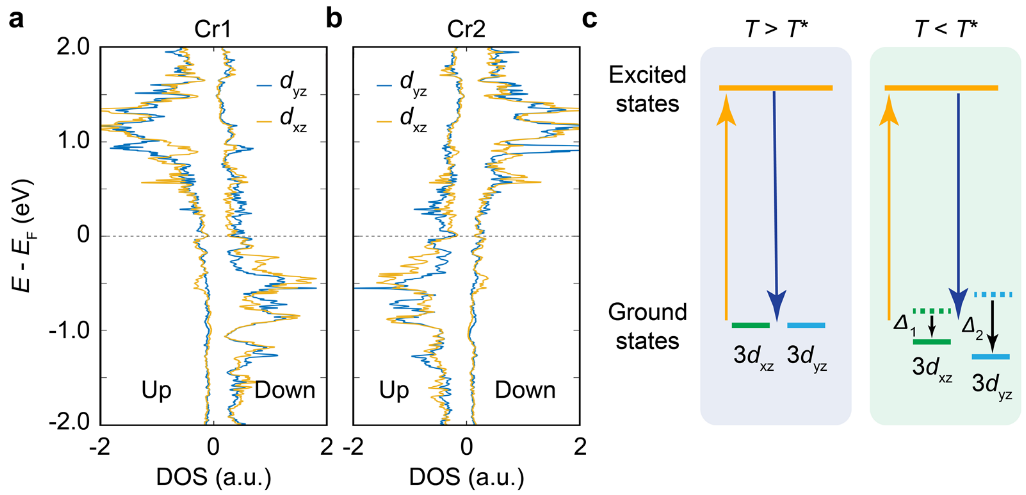

Fig. S8. (a–b) Calculated orbital-projected density of states (DOS) for the SA-ISD CDW phases. Cr1 and Cr2 atoms represent opposite spin directions, highlighting contributions from d_{yz} (blue) and d_{xz} (yellow) orbitals. (c) Schematic illustration of quasiparticle excitation and relaxation.

Figures S8a–b show the calculated orbital-projected DOS for the SA-ISD CDW phase, revealing a lifted degeneracy between the d_{yz} and d_{xz} orbitals. This non-degenerate behavior suggests the presence of an orbital order in the CDW phase. This orbital polarization likely plays a significant role in stabilizing the structure and contributing to the material's unique electronic properties. Figure S8c summarizes the whole ultrafast dynamics process, depicting the lifting of d_{xz}/d_{yz} orbitals degeneracy and the formation of an anisotropic CDW gap in the density-wave state.

11. Probe beam wavelength dependence of the TRR and TRB data in $CsCr_3Sb_5$.

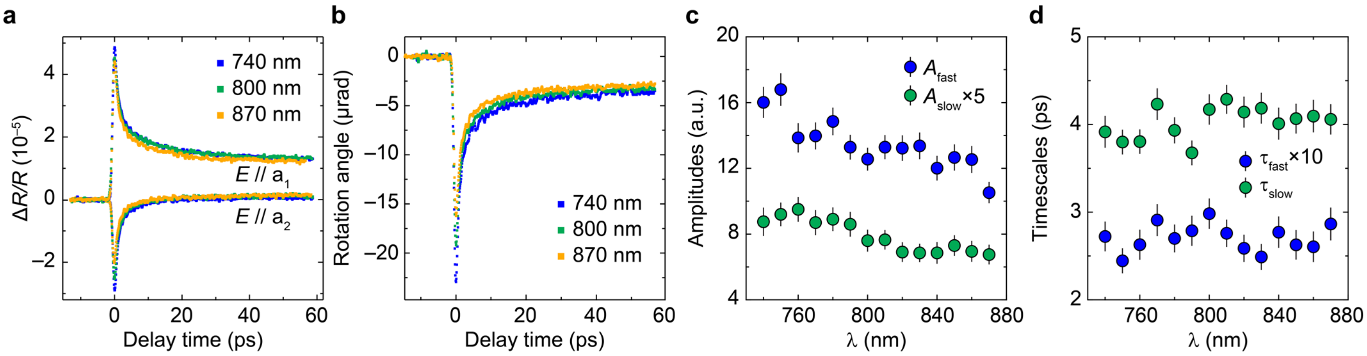

Fig. S9. Probe beam wavelength dependence of the TRR and TRB in $CsCr_3Sb_5$. (a) TRR data at selected probe beam wavelengths. The probe beam polarizations align along two principal crystal axes. (b) TRB data at selected probe beam wavelengths. (c–d), Amplitudes (c) and timescales (d) of the probe wavelength dependence of TRB data. All data were collected at $T = 3$ K. Parameters in panels (c–d) are extracted from the double-exponential fitting to the formula (1).

Figure S9 illustrates the ultrafast dynamics of both TRR and TRB data across different probe beam wavelengths. Both TRR and TRB measurements exhibit only minor changes, with decreasing amplitudes observed at longer wavelengths. The timescales remain unchanged, suggesting that the underlying physical processes are invariant to the changes in wavelength, which are primarily related to the DOS around the Fermi level. Moreover, while TRB data are presented here as a representative case, the wavelength dependence of the TRR signals follows the same trend.

12. Fitting details of the probe polarization directions and temperature dependence of the TRB data in $CsCr_3Sb_5$.

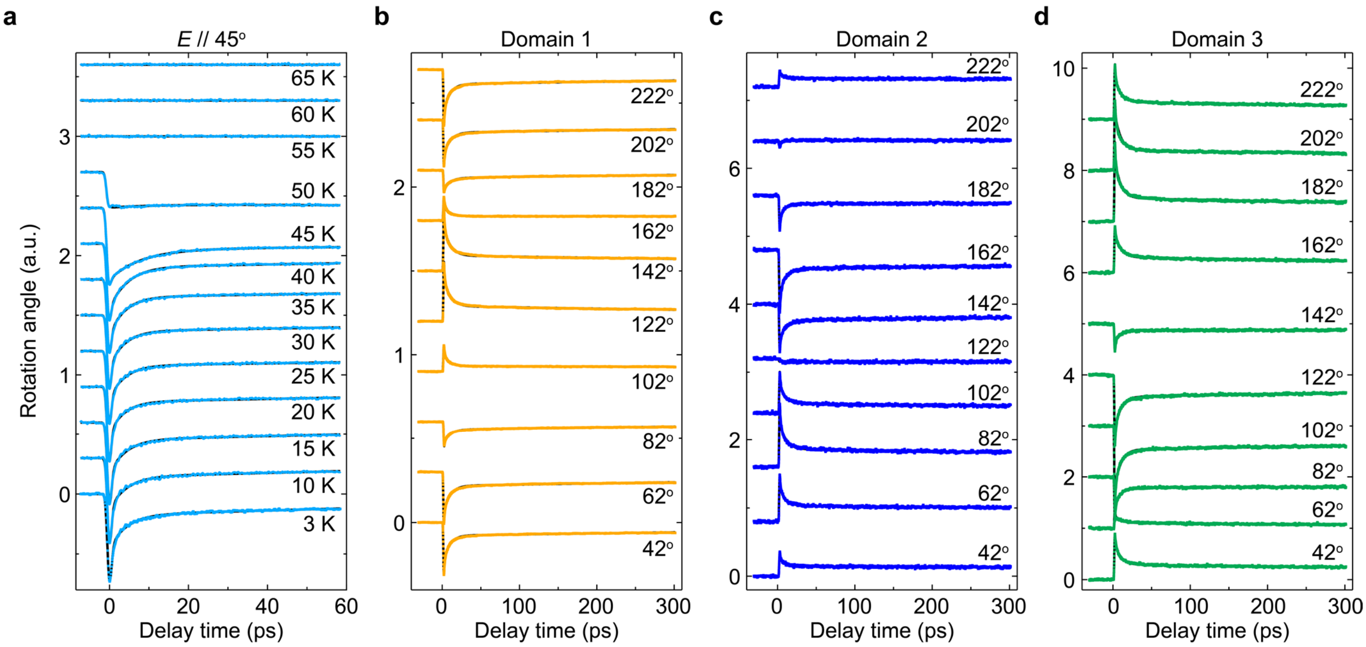

Fig. S10. Fitting details of the TRB data in $CsCr_3Sb_5$. (a) Temperature-dependent TRB data along the probe polarization direction 45° at Domain 1. (b–d), Polarization-dependent TRB data at three domains at $T = 3$ K. All fitting results overlay as black lines.

The TRB data is phenomenologically fitted to formula (1). The amplitudes $A_{\mathrm{fast}}/A_{\mathrm{slow}}$ and the relaxation timescales $\tau_{\mathrm{fast}}/\tau_{\mathrm{slow}}$ can be extracted from the transient birefringence change with this model. As shown in Figure S10, this model can nicely fit the probe polarization and temperature dependence of TRB data in Figs. 4a–c of the main text. The extracted A and τ are summarized in Figs. 4d–e of the main text.

13. Fitting details of the critical exponent of the temperature-dependent order parameter.

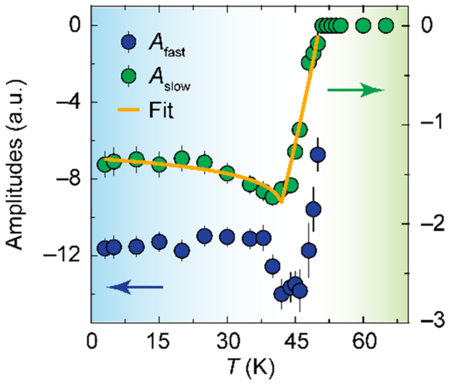

Fig. S11. Temperature-dependent amplitudes of TRB signals extracted from double-exponential fitting. The orange curve represents the fit of the critical exponent, which applies the following piecewise model.

In the equilibrium state, the individual order parameter of a phase transition, $O(T)$, can be expressed according to the Landau theory of phase transitions:

$$O(T) = \begin{cases} O_0 \left(1 - \frac{T}{T_c}\right)^{\lambda} & (T < T_c) \\ 0 & (T \geq T_c) \end{cases} \tag{9}$$

where O_0 is the order parameter at zero temperature, T_c is the critical temperature, and λ is the critical exponent characterizing the phase transition.

Birefringence serves as a direct probe of the order parameter in systems exhibiting rotational symmetry breaking. The distinct amplitudes extracted from TRB measurements are directly linked to transient changes in different order parameters. Considering a quasi-equilibrium thermal effect model, where laser excitation causes an instantaneous temperature increase ΔT, the transient birefringence amplitude $A(T)$ is proportional to the difference between $O(T)$ and $O(T + \Delta T)$ by the following piecewise model (*12*):

$$A(T) \propto \Delta O(T) = \begin{cases} O_0 \left[\left(1 - \frac{T}{T_c}\right)^{\lambda} - \left(1 - \frac{T + \Delta T}{T_c}\right)^{\lambda} \right] & (T < T_c - \Delta T) \\ O_0 \left(1 - \frac{T}{T_c}\right)^{\lambda} & (T_c - \Delta T \leq T \leq T_c) \\ 0 & (T \geq T_c) \end{cases} \tag{10}$$

This model allows for a piecewise fitting approach to describe the birefringence response as the system approaches the critical temperature T_c with a finite temperature increment ΔT induced by laser excitation, as shown in Figure S11.

The amplitude of the slow process A_{slow} can be accurately modeled by considering the perturbation of the order parameter due to the laser thermal effect (*12*), yielding a critical exponent of 0.90 ± 0.02. This value falls between the critical exponents for purely electronic and purely structural orders (*13*), but is closer to the latter, suggesting a phonon-associated CDW accompanied by a structural transition (*14*).

14. Raman spectra of the $CsCr_3Sb_5$ single crystal.

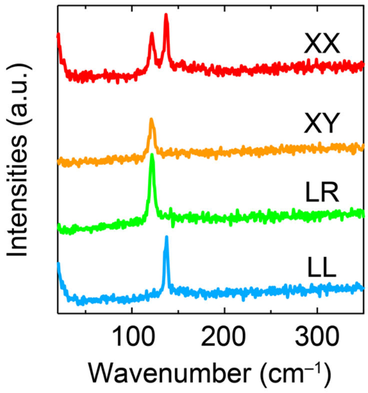

Fig. S12. Raman spectra of the $CsCr_3Sb_5$ single crystal under different polarization geometries. The input and scattered light polarizations were configured as linearly co-polarization (XX), linearly cross-polarization (XY), circularly cross-polarization (LR), and circularly co-polarization (LL), respectively.

To verify the crystal structure and phonon symmetries, we measure the high-resolution Raman spectra under different polarization geometries at T = 6 K, using a custom-built microscopy setup in a back-scattering geometry with 532 nm laser excitation. The normal incident light was focused onto the sample to a spot size of approximately 2 μm using a 50× objective, housed in a cryostat. The scattered light was collected directly by a spectrometer equipped with a liquid-nitrogen-cooled charge-coupled device (CCD) through Bragg notch filters, enabling access to the low wavenumber region. The laser power was set to 0.1 mW to prevent sample damage and minimize laser-induced heating.

We observe two phonon peaks at 120 cm^{-1} and 138 cm^{-1}, primarily from the movement of Sb atoms, which persisted at 80 K. The visibility of the peaks, determined by Raman selection rules based on symmetry, exhibit almost identical results with those observed in CsV_3Sb_5 (*15-18*). These results suggest the proper kagome crystal structure of our $CsCr_3Sb_5$ single crystal.

15. Elastoresistance measurements of $CsCr_3Sb_5$ in the modified Montgomery geometry.

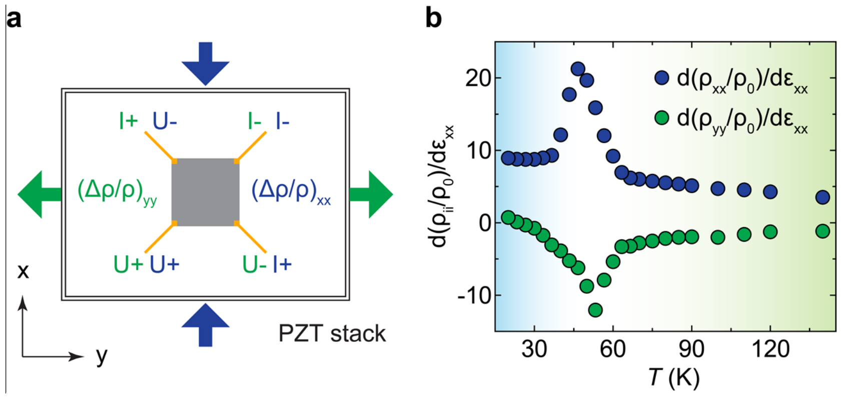

Fig. S13. Elastoresistance measurement of $CsCr_3Sb_5$ in the modified Montgomery geometry. (a) Schematic representation of the piezoelectric device and the modified Montgomery methods for elastoresistance measurement. (b) Strain-dependent relative change of resistance at different temperatures.

We observe a clear elastoresistance response near the transition temperature. The anisotropic E_{2g} component above the transition temperature follows a Curie-Weiss behavior, indicating the gradual establishment of nematic order. The nematic fluctuations peak around the transition temperature, consistent with Landau's phase transition theory. This divergent behavior is a hallmark of the electronic origin of nematicity (*19*). The Curie-Weiss behavior is fitted to the form $y = A/(x - T^*) + y_0$ for the E_{2g} response over the temperature range of 53.3–140 K (main text, Fig. 3g). The fit yields parameters $y_0 = 2.6$, $A = 78.8$, and a Curie-Weiss temperature $T^* = 48.6$ K.

References

1. V. Sunko, Y. Sun, M. Vranas, C. C. Homes, C. Lee, E. Donoway, Z. C. Wang, S. Balguri, M. B. Mahendru, A. Ruiz, B. Gunn, R. Basak, S. Blanco-Canosa, E. Schierle, E. Weschke, F. Tafti, A. Frano, J. Orenstein, Spin-carrier coupling induced ferromagnetism and giant resistivity peak in $EuCd_2P_2$. *Phys. Rev. B* **107**, 144404 (2023).
2. Y. Xu, Z. Ni, Y. Liu, B. R. Ortiz, Q. Deng, S. D. Wilson, B. Yan, L. Balents, L. Wu, Three-state nematicity and magneto-optical Kerr effect in the charge density waves in kagome superconductors. *Nat. Phys.* **18**, 1470-1475 (2022).
3. Q. Wu, Z. X. Wang, Q. M. Liu, R. S. Li, S. X. Xu, Q. W. Yin, C. S. Gong, Z. J. Tu, H. C. Lei, T. Dong, N. L. Wang, Simultaneous formation of two-fold rotation symmetry with charge order in the kagome superconductor CsV_3Sb_5 by optical polarization rotation measurement. *Phys. Rev. B* **106**, 205109 (2022).
4. Q.-Y. Wu, C. Zhang, Z.-Z. Li, W.-S. Hong, H. Liu, J.-J. Song, Y.-Z. Zhao, Y.-H. Yuan, B. Chen, X.-Q. Ye, S. Li, J. He, H. Y. Liu, Y.-X. Duan, H.-Q. Luo, J.-Q. Meng, Hidden nematic fluctuation in the triclinic $(Ca_{0.85}La_{0.15})_{10}(Pt_3As_8)(Fe_2As_2)_5$ superconductor revealed by ultrafast optical spectroscopy. *Phys. Rev. B* **108**, 205136 (2023).
5. K.-H. Lin, K.-J. Wang, C.-C. Chang, Y.-C. Wen, D.-H. Tsai, Y.-R. Wu, Y.-T. Hsieh, M.-J. Wang, B. Lv, P. C.-W. Chu, M.-K. Wu, Observation of pseudogaplike feature above T_c in LiFeAs by ultrafast optical spectroscopy. *Phys. Rev. B* **90**, 174502 (2014).
6. Y. C. Tian, W. H. Zhang, F. S. Li, Y. L. Wu, Q. Wu, F. Sun, G. Y. Zhou, L. Wang, X. Ma, Q.-K. Xue, J. Zhao, Ultrafast Dynamics Evidence of High Temperature Superconductivity in Single Unit Cell FeSe on$SrTiO_3$. *Phys. Rev. Lett.* **116**, 107001 (2016).
7. P. G. Klemens, Anharmonic Decay of Optical Phonons. *Phys. Rev.* **148**, 845-848 (1966).
8. C. Xu, S. Wu, G.-X. Zhi, G. Cao, J. Dai, C. Cao, X. Wang, H.-Q. Lin, Altermagnetic ground state in distorted Kagome metal $CsCr_3Sb_5$. *Nat. Commun.* **16**, 3114 (2025).
9. F. Xie, Y. Fang, Y. Li, Y. Huang, L. Chen, C. Setty, S. Sur, B. Yakobson, R. Valentí, Q. Si, Electron correlations in the kagome flat band metal $CsCr_3Sb_5$. *Phys. Rev. Res.* **7**, L022061 (2025).
10. Y. Wang, *arXiv: 2401.16770* (2024).
11. S. Wu, C. Xu, X. Wang, H.-Q. Lin, C. Cao, G.-H. Cao, Flat-band enhanced antiferromagnetic fluctuations and superconductivity in pressurized $CsCr_3Sb_5$. *Nat. Commun.* **16**, 1375 (2025).
12. A. Zong, Q. Zhang, F. Zhou, Y. Su, K. Hwangbo, X. Shen, Q. Jiang, H. Liu, T. E. Gage, D. A. Walko, M. E. Kozina, D. Luo, A. H. Reid, J. Yang, S. Park, S. H. Lapidus, J. H. Chu, I. Arslan, X.

Wang, D. Xiao, X. Xu, N. Gedik, H. Wen, Spin-mediated shear oscillators in a van der Waals antiferromagnet. *Nature* **620**, 988-993 (2023).

13. J. W. Harter, Z. Y. Zhao, J.-Q. Yan, D. G. Mandrus, D. Hsieh, A parity-breaking electronic nematic phase transition in the spin-orbit coupled metal $Cd_2Re_2O_7$. *Science* **356**, 295-299 (2017).
14. Y. Liu, Z.-Y. Liu, J.-K. Bao, P.-T. Yang, L.-W. Ji, S.-Q. Wu, Q.-X. Shen, J. Luo, J. Yang, J.-Y. Liu, C.-C. Xu, W.-Z. Yang, W.-L. Chai, J.-Y. Lu, C.-C. Liu, B.-S. Wang, H. Jiang, Q. Tao, Z. Ren, X.-F. Xu, C. Cao, Z.-A. Xu, R. Zhou, J.-G. Cheng, G.-H. Cao, Superconductivity under pressure in a chromium-based kagome metal. *Nature* **632**, 1032-1037 (2024).
15. S. Wu, B. R. Ortiz, H. Tan, S. D. Wilson, B. Yan, T. Birol, G. Blumberg, Charge density wave order in the kagome metal AV_3Sb_5 (A = Cs, Rb, K). *Phys. Rev. B* **105**, 155106 (2022).
16. D. Wulferding, S. Lee, Y. Choi, Q. Yin, Z. Tu, C. Gong, H. Lei, S. Yousuf, J. Song, H. Lee, T. Park, K.-Y. Choi, Emergent nematicity and intrinsic versus extrinsic electronic scattering processes in the kagome metal CsV_3Sb_5. *Phys. Rev. Res.* **4**, 023215 (2022).
17. G. Liu, X. Ma, K. He, Q. Li, H. Tan, Y. Liu, J. Xu, W. Tang, K. Watanabe, T. Taniguchi, L. Gao, Y. Dai, H.-H. Wen, B. Yan, X. Xi, Observation of anomalous amplitude modes in the kagome metal CsV_3Sb_5. *Nat. Commun.* **13**, 3461 (2022).
18. F. Jin, W. Ren, M. Tan, M. Xie, B. Lu, Z. Zhang, J. Ji, Q. Zhang, π Phase Interlayer Shift and Stacking Fault in the Kagome Superconductor CsV_3Sb_5. *Phys. Rev. Lett.* **132**, 066501 (2024).
19. J.-H. Chu, H.-H. Kuo, J. G. Analytis, I. R. Fisher, Divergent Nematic Susceptibility in an Iron Arsenide Superconductor. *Science* **337**, 710 (2012).
